# Bilateral Focused Ultrasound Pallidotomy for Parkinson‐Related Facial Dyskinesia—A Case Report

**DOI:** 10.1002/mdc3.13462

**Published:** 2022-05-09

**Authors:** Lennart H. Stieglitz, Sujitha Mahendran, Markus F. Oertel, Christian R. Baumann

**Affiliations:** ^1^ Department of Neurosurgery University Hospital Zurich Zurich Switzerland; ^2^ Department of Neurology University Hospital Zurich Zurich Switzerland

**Keywords:** HiFUS, pallidotomy, Parkinson

## Abstract

**Background:**

For safety reasons, both magnetic resonance‐guided high‐intensity focused ultrasound (MRgHiFUS) thalamotomy and pallidotomy are currently approved exclusively for unilateral treatment, but axial symptoms like levodopa‐induced orofacial dyskinesia require a bilateral approach.

**Objectives:**

We report the first case of successful bilateral MRgHiFUS pallidotomy for peak‐dose dyskinesia in a patient with Parkinson's disease (PD).

**Methods:**

The treatment decision was based on the patient's reluctance toward brain implants and pump therapies and the fact that he had limited access to a deep brain stimulation center in his home country. The treatment was planned as staged procedure with an interval of 18 months because of travel restrictions because of the coronavirus disease (COVID)‐19 pandemic.

**Results:**

After the second treatment, levodopa‐induced orofacial dyskinesia remitted and improved bradykinesia and rigidity with stable gait and good postural reflexes.

**Conclusions:**

This promising result suggests that in selected PD patients with dyskinesia, staged bilateral MRgHiFUS pallidotomy might be considered.

Magnetic resonance‐guided high‐intensity focused ultrasound (MRgHiFUS) is still a relatively new treatment option for movement disorders. The Food and Drug Administration (FDA) approved MRgHiFUS thalamotomy in July 2016 for the treatment of essential tremor, and in December 2018, an approval for treatment of tremor‐dominant Parkinson's disease (PD) followed. Only very recently, in November 2021, the method gained an expansion of FDA approval to target the globus pallidus internus (GPi) to treat other PD symptoms including bradykinesia, rigidity, and dyskinesia. For safety reasons, both MRgHiFUS thalamotomy and pallidotomy are currently approved exclusively for unilateral treatment. We report the first case of a bilateral MRgHiFUS based pallidotomy for the treatment of disabling axial levodopa (l‐dopa)‐induced dyskinesia in a patient suffering from PD in the form of a named patient use.

## Methods

### Case Description

We report the case of a 77‐year‐old Jordanian male patient. He was diagnosed with PD in 2018. After earlier onset of prodromal symptoms including hyposmia and rapid eye movement (REM) sleep behavior disorder, first motor symptoms including bradykinesia, and mild tremor started in 2016, together with mild depression and loss of weight because of reduced appetite. Immediately after diagnosis, treatment with l‐dopa‐carbidopa and amantadine was started, with satisfactory improvement of bradykinesia. However, in early 2020, disabling perioral peak‐dose dyskinesia occurred, leading to the introduction of amantadine that was later discontinued again because of loss of efficacy. Furthermore, he suffered from wearing‐off 3–4 h after l‐dopa intake.

Escitalopram improved his mood, and donepezil was prescribed because of cognitive impairment.

In our outpatient clinic at admission in March 2020, the Hoehn and Yahr scale was 2.5 and total l‐dopa dose was 800 mg per day. Medication was adapted several times, from 3 × 250 mg l‐dopa to 4 × 200 mg l‐dopa because of peak‐dose dyskinesia, then by us to 4 × 100 mg l‐dopa and finally to 3–6 × 75 mg l‐dopa, again because of disturbing dyskinesia. With 225 mg l‐dopa per day, dyskinesia was much milder, but bradykinesia became more burdensome. During a l‐dopa challenge test, the Movement Disorder Society‐Sponsored Revision of the Unified Parkinson's Disease Rating Scale (MDS‐UPDRS) III was 43 points in medication off condition, with general right‐dominant bradykinesia, marked hypokinesia in repetitive movements, rigidity, intermittent resting tremor of the right hand, reduced swing of the right arm, hypomimia, and hypophonia. Sixty minutes after intake of 300 mg l‐dopa, a higher‐than‐regular dose, the MDS‐UPDRS III was at 27 points. On medication, ambulation and voice were clearly improved, but perioral dyskinesia persisted for more than an hour and significantly reduced his ability to speak (see Video 1). As a first measure, we reduced both l‐dopa doses and intervals between medication intake, which mildly improved his general condition and mood, but pronounced perioral peak‐dose dyskinesia persisted. The patient was not satisfied with this outcome and asked for therapeutic alternatives, in particular to reduce the perioral movements. Given the fact that the patient lived in Jordan, without sufficient access to a deep brain stimulation (DBS) center and given his reluctance toward both brain implants and pump therapies, we discussed the option of experimental pallidal MRgHiFUS. We openly declared that axial dyskinesia most probably necessitates bilateral treatment and started extensive evaluations to check the suitability of the patient for this procedure.

**Video 1:** The video shows the patient's condition (1) during baseline, (2) before the second pallidotomy, and (3) after the second pallidotomy. The patient is shown in peak‐dose‐condition during all examinations.

## Results

### Pre‐Interventional Assessments

The magnetic resonance imaging (MRI) revealed vascular leucencephalopathy Fazekas grade 1 but no other relevant pathological findings. Simulation of sonic properties of the patient's skull in computed tomography (CT) scan revealed a skull‐density ratio (SDR) of 0.39, and therefore, the patient was found to be eligible for MRgHiFUS treatment. Neuropsychological evaluation showed fronto‐cortically reduced functions, the Montréal Cognitive Assessment was at 20/30 points. Psychiatric evaluation revealed a mild symptomatic depression related to PD. Reduction of mobility and consecutive loss of autonomy contributed to mood problems, but posed no contraindication for surgical treatment escalation. Pre‐interventional *off*‐med MDS‐UPDRS III during l‐dopa challenge test was 43 and 27 with isolated yet marked perioral dyskinesia on medication (60 min after 200 mg l‐dopa) (Video 1). We discussed the case intensely during the institutional interdisciplinary and interprofessional board for movement disorders therapies and assumed eligibility for staged bilateral pallidal ablation. We again thoroughly informed about the experimental character of bilateral MRgHiFUS pallidotomy, the unproven benefits and potential risks, the expectation that only bilateral treatment might improve perioral dyskinesia and the fact that such bilateral treatment could only be achieved in a staged procedure.

### Bilateral Pallidotomy

**FIG. 1:**
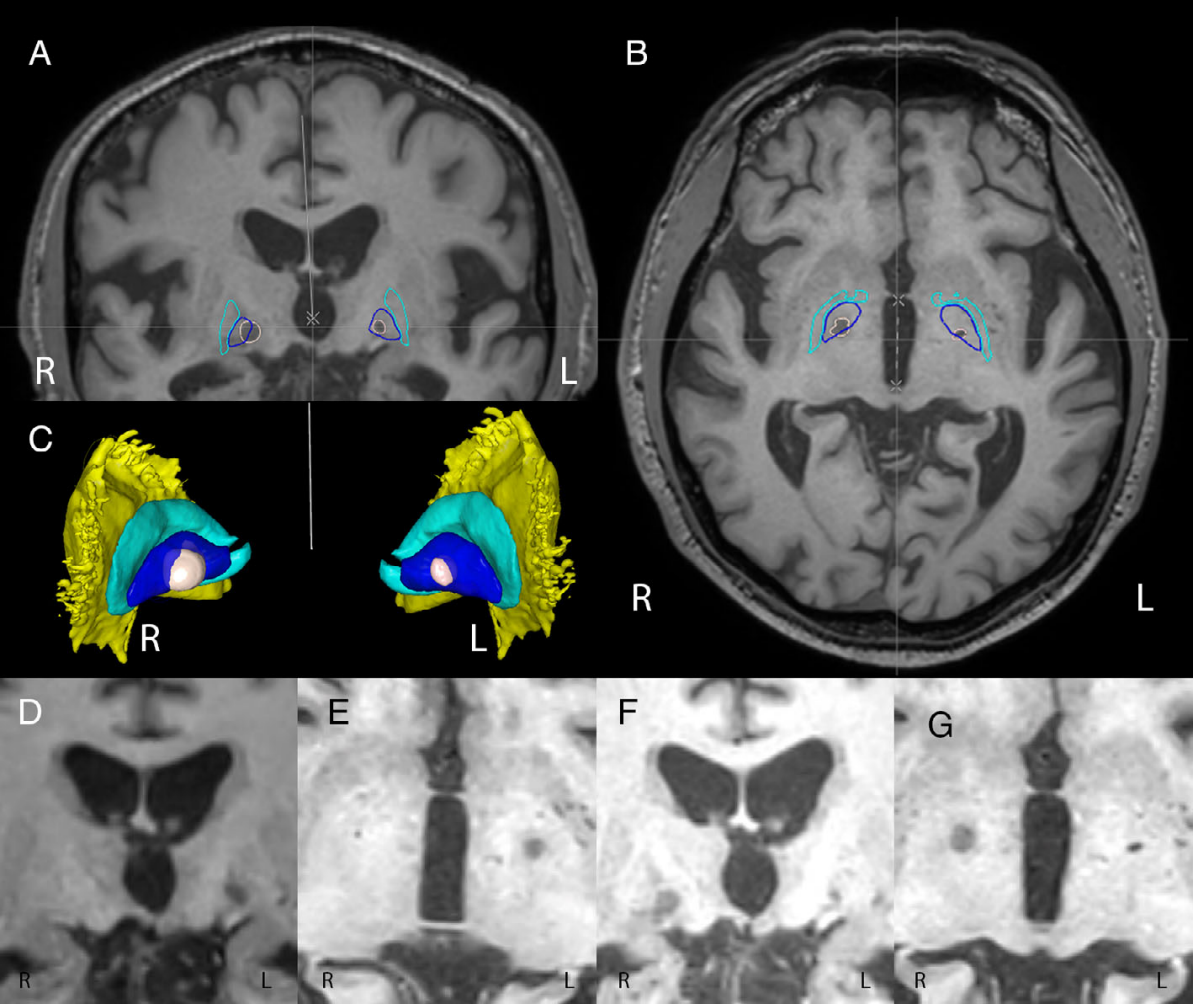
Cranial MRI after bilateral pallidotomies in coronar (**A**), axial (**B**) and 3D (**C**). The ablations are outlined in beige. For better orientation, a 3D‐atlas was superposed using Medtronic SureTune 3.0 (Medtronic, Minneapolis, MN). The GPi is outlined in dark blue, Globus pallidus externus (GPe) in light blue and putamen in yellow (only 3D image). The images (**D**) and (**E**) show the fresh left‐sided ablation in the first day postoperatively; the images (**F**) and (**G**) show the fresh right‐sided ablation on the first day postoperatively. In all images, letters R and L indicate laterality.

The staged MRgHiFUS pallidotomy was performed in May 2020 (left pallidotomy) and November 2021 (right pallidotomy). The originally planned interval of 6 months had to be prolonged because of travel restrictions during the coronavirus disease (COVID)‐19 pandemic. Anterior to posterior commissure (AC‐PC) distance was 26 mm and width of third ventricle was 11 mm. The left GPi was targeted at x = 20.1 mm, y = 9.2 mm posterior, z = 3.7 mm inferior, the right GPi at x = 20 mm, y = 9.5 mm posterior, z = 3.5 mm inferior of the mid‐commissural point related to AC‐PC. The first ablation was performed with three therapeutic sonications at 26500 J maximum power and 59°C maximum target temperature. The second ablation required five sonications with therapeutic intentions, of which two were automatically aborted because of tissue cavitations. Three sonications at 20,000 J maximum power reached 58°C maximum target temperature. Peri‐interventional neurological exams revealed no new neurological deficits. During the first treatment, the patient reported mild headache during the high‐power sonications. Figure 1 shows the MRI 24 h after the second treatment with bilateral GPi lesions, the left 18 months old and the right 1 day old with perilesional edema.

### Post‐Interventional Result

After unilateral treatment, the patient reported neither discomfort nor side effects, and MD‐UPDRS III in a habitual ON, (ie, on regular medication at an arbitrary time point [here, 140 min after 250 mg l‐dopa]) was 38. Perioral dyskinesia was slightly reduced. Additionally, at thorough neurological examinations, we identified no new neurological deficits. The patient returned home to Jordan after the first treatment. During the flight, he suffered from a deep venous thrombosis in the leg after forgotten intake of his antithrombotic medication with dabigatran. Back in Jordan and after this incident, he was in a reduced general condition and treated with short‐acting l‐dopa‐carbidopa (5 × 100 mg), pramipexole, and again amantadine, the latter in the attempt to reduce perioral dyskinesia, which returned to pre‐MRgHiFUS condition. The patient and his relatives, however, reported that reduction of tremor and improvement of movements persisted on the right side after left‐sided MRgHiFUS. After the second treatment, now in the right GPi, we installed a dose‐reduced medication with dual release l‐dopa‐benserazid (375 mg per day, ie, 5 × 75 mg), and continued pramipexole and amantadine. The position and size of the lesions are shown in Figure 1. One week after treatment, the patient suffered from no orofacial dyskinesia anymore (Video 1). MDS‐UPDRS III *on* medication (75 min after 75 mg l‐dopa) was 15, with no tremor and only mild bilateral bradykinesia and rigidity, stable gait, and good postural reflexes. The latest follow‐up so far was 2.5 months after the second treatment. There were no pallidotomy‐related side effects and walking was stable with no balance and gait‐disorder. REM sleep disorder improved after second treatment. The patient showed no cognitive decline and no negative impact on his psyche.

## Discussion

The recent publications by Martínez‐Fernández et al,^1^ Iorio‐Morin et al,^2^ and Fukutome et al^3^ reported encouraging results after staged bilateral MRgHiFUS thalamotomy in essential tremor patients. These observations indicate that staged bilateral ablations with MRgHiFUS might be possible and safe. Another publication by Martínez‐Fernández et al^4^ showed the clear motor improvement of PD patients after unilateral MRgHiFUS subthalamotomy. A pilot study about bilateral subthalamotomy in PD (NCT03964272) should be concluded in the near future and one randomized controlled study is in preparation. Eisenberg et al^5^ recently published a series of 20 successful unilateral MRgHiFUS pallidotomies for PD patients suffering from dyskinesias. Axial symptoms, however, are far more challenging to treat because they usually do not respond to unilateral stimulation or ablation. Treatment chances and risks must be weighed carefully against each other, but long‐time experience from bilateral DBS in the GPi suggests that the profile of expected side effects should be acceptable.^6^ Gallay et al^7^ reported a series of 52 unilateral pallidothalamic tract (PTT)‐ablations in 2020 with good effectiveness on dyskinesias and favorable risk‐profile, and there is one ongoing open‐label study by Insightec about bilateral PTT ablation for the treatment of PD (NCT04728295).

The presented case would have qualified for bilateral pallidal DBS, less so for subthalamic DBS because of the cognitive deficits already present at first evaluation. Fortunately, the main goal of the treatment was reduction of the disabling orofacial peak‐dose dyskinesia, therefore, targeting the GPi was considered appropriate irrespective of the technique applied, DBS, or MRgHiFUS. The patient's clear decision against DBS and the limited access to continued neurological treatment with DBS experience at the patient's home were strong arguments for probing staged bilateral MRgHiFUS. In this—to our knowledge first case—staged bilateral MRgHiFUS pallidotomy completely resolved the disturbing axial peak‐dose dyskinesia and improved the overall motor function despite reduction of the l‐dopa dose, without any ablation‐related treatment side‐effects. This promising result suggests that in selected PD patients with dyskinesia, staged bilateral MRgHiFUS pallidotomy might be considered. Still, previous publications from bilateral radiofrequency‐pallidotomies, such as the series published by Parkin et al^8^ in 2002, reported cases of choroathetoid dyskinesia, deterioration of speech, salivation, on‐state gait freezing, and handwriting. Safety and long‐term effects, should, therefore, be thoroughly studied in appropriate prospective trials.

## Author Roles

(1) Research project: A. Conception, B. Organization, C. Execution; (2) Statistical analysis: A. Design, B. Execution, C. Review and Critique; (3) Manuscript: A. Writing the First Draft, B. Review and Critique.

L.S.: 1A, 1B, 1C, 3A

S.M.: 1C, 3B

M.O.: 1C, 3B

C.B.: 1A, 1B, 1C, 3B

## Disclosures

### Ethical Compliance Statement

The authors confirm that the approval of an institutional review board was not required for this work. The patient provided his informed consent before treatment. We confirm that we have read the Journal's position on issues involved in ethical publication and affirm that this work is consistent with those guidelines.

### Funding Sources and Conflicts of Interest

The authors declare that there are no funding sources or conflicts of interest relevant to this work.

### Financial Disclosures for the Previous 12 Months

L.H.S. received speaker fees from Admirabiles, Insightec, Brainlab, and Neuromedex and declares that there are no additional disclosures to report. S.M. declares that there are no additional disclosures to report. M.F.O. declares that there are no additional disclosures to report. C.R.B. received speaker fees from Novartis Pharma and AbbVie Pharma and he received grants from AbbVie Pharma, GE Healthcare, Roche Pharma, and Medtronic. He declares that there are no additional disclosures to report.
